# Pharmacokinetics, pharmacodynamics and safety of 15 mg-tolvaptan administered orally for 7 consecutive days to Chinese patients with child-Pugh B cirrhosis

**DOI:** 10.3389/fphar.2024.1324299

**Published:** 2024-01-26

**Authors:** Hongzhong Liu, Yongfeng Wang, Tao Liu, Yingxuan Chen, Xin Zheng, Ming Liu, Qian Zhao, Minde Zeng, Ji Jiang, Yimin Mao, Pei Hu

**Affiliations:** ^1^ Clinical Pharmacology Research Centre, Peking Union Medical College Hospital, Chinese Academy of Medical Sciences & Peking Union Medical College, Beijing Key Laboratory of Clinical PK and PD Investigation for Innovative Drugs, Beijing, China; ^2^ Division of Gastroenterology and Hepatology, Shanghai Institute of Digestive Disease, Renji Hospital, School of Medicine, Shanghai Jiao Tong University, Shanghai, China

**Keywords:** tolvaptan, pharmacokinetics, pharmacodynamics, child-Pugh B cirrhosis, ascites, Chinese adult patients

## Abstract

**Background:** Tolvaptan, a selective vasopressin V_2_-receptor antagonist, can elicit a diuretic effect without significant electrolyte loss. The aims were to evaluate multiple-dose pharmacokinetics, pharmacodynamics and safety of daily administration of 15 mg tolvaptan in Chinese adult patients with confirmed Child-Pugh Class B cirrhosis accompanied by ascites.

**Methods:** This was an open-label, single-center, single- and multiple-dose study. All patients received a daily 15 mg dose of tolvaptan for 7 consecutive days. The plasma concentrations of tolvaptan and its two metabolites (DM-4103, DM-4107) were measured using high-performance liquid chromatography with tandem mass spectrometry (HPLC-MS/MS). In addition, various pharmacokinetics parameters were calculated. The pharmacodynamic outcomes evaluated changes in serum sodium and potassium concentrations, daily urine volume, daily water consumption, fluid balance and body weight. Safety profiles, including the incidence of treatment-emergent adverse events (TEAEs), were carefully recorded.

**Results:** Eleven patients with Child-Pugh B cirrhosis were eventually enrolled in the study. Plasma concentrations of tolvaptan and DM-4107 reached steady-states after 7 days of consecutive oral administration. No accumulation of tolvaptan or DM-4107 was found, but DM-4103 accumulated 18.2-fold after multiple-dosing. The daily urine volume and daily water consumption were statistically significantly increased after administration of tolvaptan from Day 1 to Day 7 (all *p* < 0.05), accompanied by an increased serum sodium concentration. Of 11 patients, 9 (81.8%) reported 20 TEAEs, with the majority being mild to moderate in severity. The most commonly occurring TEAEs were thirst (45.5%), pollakiuria (36.4%) and dry mouth (27.3%).

**Conclusion:** Tolvaptan at a daily dose of 15 mg had a diuretic effect but did not increase serum sodium excretion or lead to tolvaptan accumulation. It is therefore can be safely used for short-term treatment of Chinese adult patients with confirmed Child-Pugh B cirrhosis.

**Clinical Trial Registration:** https://clinicaltrials.gov/search?term=NCT01359462, identifier NCT01359462.

## 1 Introduction

Tolvaptan is an orally active, selective vasopressin V_2_-receptor antagonist that has been approved for the treatment of clinically significant hypervolemic and euvolemic hyponatremia for patients with heart failure and the syndrome of inappropriate antidiuretic hormone. It is given in the United States, Europe, Japan and China, at an initial recommended dose of 15 mg/d (Schrier et al., 2006; Dubois et al., 2012). The pharmacokinetic (PK) properties of tolvaptan have been characterized in healthy Caucasian, Japanese and Korean subjects. Tolvaptan reaches a maximum plasma concentration 2–4 h after a single-dose and no accumulation was observed after multiple-doses (Shoaf et al., 2007; Kim et al., 2011; Yi et al., 2012). In addition, food did not affect the PK profiles of tolvaptan compared with the fasted state (Kim et al., 2011; Shoaf et al., 2012a). As CYP3A4 is the primary enzyme responsible for the metabolism of tolvaptan, co-administration of the CYP3A4 inhibitor-ketoconazole in healthy subjects resulted in a 3.48-fold higher peak concentration (C_max_) and 5.40-fold higher area under curve (AUC), respectively (Shoaf et al., 2012b). Tolvaptan is metabolized almost exclusively by CYP3A and 14 metabolites have been identified in plasma, urine and feces. Apart from one they were also metabolized by CYP3A and none of the derivatives were pharmacologically active. After oral administration of radiolabeled tolvaptan, tolvaptan was a minor component in plasma, representing just 3% of the total plasma radioactivity. The oxobutyric acid metabolite (DM-4103) was present as 52.5% of total plasma radioactivity, with all other metabolites present at lower concentrations than tolvaptan. About 40% of the radioactivity was recovered in the urine (< 1% as unchanged tolvaptan) and 59% in feces (19% as unchanged tolvaptan) (Otsuka, 2022).

Compared with traditional regimens, tolvaptan promotes free water excretion without significant electrolyte loss by blocking selectively the V_2_ receptor in the principal cells of the collecting duct thus enhancing the diuretic effect (Hirano et al., 2000). Several studies have reported that tolvaptan was effective in cirrhotic ascites patients who could not be treated with doses of conventional diuretics, due to electrolyte abnormalities or large doses of diuretics and salt intake restriction (Sakaida et al., 2013; Okita et al., 2014; Sakaida et al., 2014). Accordingly, tolvaptan-7.5 mg/d was approved for the treatment of hepatic edema in Japan in 2013, providing an effective regimen to treat ascites induced by cirrhosis (Sakaida et al., 2020). Tolvaptan has not been approved in China for the treatment of cirrhotic ascites, but two clinical trials were conducted in these Chinese patients from March 2009 to February 2010, and from October 2010 to January 2012, respectively (Wang et al., 2018; Tang et al., 2020). One phase 2 trial showed that tolvaptan at 15 or 30 mg/d significantly decreased the body weight and abdominal circumference compared with placebo, but without a significant difference between the tolvaptan-treated groups (Wang et al., 2018). A phase 3 trial showed that either 7.5 mg or 15 mg of tolvaptan resulted in a significant weight loss compared to placebo. Except for a greater 24-h cumulative urine volume in the tolvaptan-15 mg group, all other efficacy indicators were not significantly different (Tang et al., 2020). Accordingly, Chinese guidelines on the management of ascites in cirrhosis recommend that tolvaptan be used for the treatment of patients with grade 2/3 ascites, recurrent ascites, or those who have poor responses to traditional diuretics regimens, with an initial dose of 7.5–15 mg/d (Chinese Society of Hepatology and Chinese Medical Association, 2023). However, the greatest concern is the risk for irreversible and fatal liver damage from tolvaptan that was reported in a phase 3 clinical trial conducted in patients with autosomal dominant polycystic kidney disease (ADPKD) (Torres et al., 2012). Subsequently, an independent, blinded expert liver adjudication committee reviewed the data and found that patients who experienced hepatocyte damage events received high doses of oral tolvaptan (120 mg/day) for more than 3 months (Watkins et al., 2015). Furthermore, no lopsided hepatocyte damage events were found in patients with hyponatremia, heart failure or cirrhosis who had been treated with a lower-dose of tolvaptan compared to placebo. Thus, Chinese and FDA guidelines both recommend that tolvaptan be used continuously for no more than 30 days to minimize the risk of liver damage.

Based on this background research, an open-label, single-center, single- and multiple-dose study was conducted in Chinese patients with confirmed Child-Pugh B cirrhosis who may have accompanied ascites. The main aim was to investigated the PK characteristics of tolvaptan following administration of a single and multiple 15-mg oral doses, followed by the exploration of its pharmacodynamics (PD) and safety properties.

## 2 Methods

### 2.1 Study design and patients

The open-label, single center, single- and multiple-dose study aimed to evaluate PK, PD and safety profiles of 15-mg tolvaptan tablets in Chinese adult patients with Child-Pugh B cirrhosis (accompanied by ascites). Patients were orally administered 15 mg tolvaptan once daily for 7 consecutive days. Patients abstained from food after 10 p.m. every evening and tolvaptan was orally administered the next morning after breakfast. This study was conducted from April to November in 2009 in accordance with the Declaration of Helsinki and Good Clinical Practice guidelines of the International Conference on Harmonization. Study protocols and informed consent forms were reviewed and approved by the independent ethics committee of Renji Hospital (Approval No. RJ-EC [2009] 12). Written informed consent was obtained from each patient before enrollment. The trial was registered with ClinicalTrials.gov (identifier: NCT01359462).

Hospitalized hepatocirrhosis patients who were diagnosed with Child-Pugh B (scores 7–9, likely accompanied by ascites), were eligible to participate in the study if they were aged between 18 and 75 years and able to sign informed consent. Key exclusion criteria were: patients with hepatic encephalopathy (coma degree classification ≥ Grade 2); likely to have gastrointestinal bleeding during the study period; malignant ascites; heart failure (NYHA Class III and IV); spontaneous bacterial peritonitis; anuria (daily urine output < 100 mL); and dysuria induced by urinary tract stenosis, calculus and tumor. In addition, patients with a systolic blood pressure < 90 mmHg, a serum creatinine concentration > 2.5 times the upper limit of the normal range, serum Na^+^ > 145 mmol/L (or higher than the upper limit of the normal range), serum K^+^ > 5.5 mmol/L, uric acid > 476 μmol/L, or other unfavorable conditions were also excluded.

### 2.2 PK determinations

The PK outcomes were the plasma concentrations and PK parameters of tolvaptan, DM-4103 and DM-4107 after single dosing on Day 1 and after multiple dosing on Day 7. Moreover, the trough concentrations of tolvaptan, DM-4103 and DM-4107 at 24 h after 1-day, 5-day, 6-day and 7-day oral administration were also measured. PK parameters including the AUC, C_max_, time to reach C_max_ (t_max_), terminal half-life (t_1/2_), apparent clearance (CL/F), apparent volume of distribution (V_z_/F) and accumulation ratio (R_AC_), after a single dose and multiple dosing were determined, if applicable.

Following tolvaptan administration, plasma samples were collected at the following time points: pre-dose, 2, 4, 6, 8, 12 and 24 h after a single-dose on Day 1, the pre-dose on Day 6 and Day 7, 2, 4, 6, 8, 12 and 24 h after the last administration on Day 7.

The plasma concentration of tolvaptan, DM-4103, and DM-4107 were analyzed by a fully validated bioanalytical method using a high-performance liquid chromatography system with tandem mass spectrometry (HPLC-MS/MS), with the lower limit of quantification being 1.00 ng/mL for tolvaptan, DM-4103 and DM-4107. Calibration, quality control, and clinical samples (100 μL) were spiked with 150 μL OPC-41100 (40 ng/mL) methanol solution as an internal standard and processed by a solid phase extraction method (OASIS HLB (30 mg) SPE Plate). Eluent (800 µL) was evaporated to dryness and reconstituted in 100 μL of methanol-water = 1:2 (v/v); 30 μL were injected and simultaneously assayed for tolvaptan, DM-4103 and DM-4107 using reversed-phase HPLC with Turbo Ion Spray^®^ Sciex (Framingham, MA) tandem mass spectrometric detection. The analytes were separated by Waters Symmetry C18 4.6 mm × 50 mm (3.5 µm) at 40°C using methanol-water-formic acid = 70:30:0.25 (v/v/v) at a flow rate of 0.8 mL/min under isocratic conditions. The total run time was 3.5 min. Multiple reaction monitoring of analytes was conducted in the positive mode at m/z 449.1 to 252.2, 479.0 to 252.2, 481.0 to 119.2, and 463.1 to 266.2 for tolvaptan, DM-4103, DM-4107 and the internal standard, respectively. Analyte-to-internal standard peak area ratios for the standards were used to create a calibration curve using 1/x^2^ weighted least-squares regression analysis.

### 2.3 PD evaluation

The PD outcomes included changes in serum sodium and potassium concentrations, daily urine volume, daily water consumption, fluid balance and pre-meal body weight over 7 days of continuous oral administration of tolvaptan.

Serum samples were collected from Day 1 to Day 8 before dosing and 12 h after 1-day and 7-day oral administration of tolvaptan for the measurement of serum sodium and potassium concentrations. Daily urine volume and daily water consumption were recorded once from Day −1 (screening period) after breakfast to Day 8 before breakfast, with an interval of 24 h. After breakfast, all patients emptied their bladders before drug administration. Daily water consumption included water intake, water contained in food, water injection, etc. Fluid balance was defined as the difference between the daily urine volume and daily water consumption (fluid balance = daily urine volume - daily water consumption). Changes in pre-meal body weight were recorded from Day 1 to Day 8 before each dose.

### 2.4 Safety assessment

Safety outcomes were the incidence and severity of treatment-emergent adverse events (TEAEs), drug-related TEAEs or serious AEs (SAEs). TEAE was defined as an event that occurred during treatment, having been absent in the pre-treatment period, or worsened relative to the pre-treatment state. Drug-related TEAEs included those that were certain, probable or possibly associated with tolvaptan treatment. Furthermore, laboratory examinations (routine blood/urine analysis, blood biochemistry, coagulation index), vital signs (blood pressure, pulse and body temperature), physical examination, 12-lead electrocardiogram (ECG) were also assessed.

Laboratory examination results and the 12-lead ECG were assessed on the screening day (Day −1) and 24 h after 7-day oral administration of tolvaptan. Vital signs and physical examinations were conducted on Day 1 to Day 8 before each dose of tolvaptan was administered. Measurements of blood pressure, pulse and physical examinations were also made 4 h, 8 h and 12 h after 1-day and 7-day oral administration.

### 2.5 Statistical analysis

All statistical analyses were performed using SAS software (version 9.1.3, SAS Institute Inc., Cary, United States). Continuous variables are presented as the mean ± standard deviation (SD) or medians with range (minimum, maximum), while categorical variables are given as numbers with percentages. The comparison between daily urine volume, daily water consumption and fluid balance at the 7-day treatment time point and baseline were performed using a paired rank sum test. A paired *t*-test was employed to compare the significance of the remaining continuous variables from baseline with each treatment time point. All statistical tests were conducted using a two-sided test, and a *p*-value < 0.05 was considered to be statistically significant. The plasma concentration below the LLOQ was recorded as 0 when calculating the mean plasma concentration for tolvaptan, DM-4103 and DM-4107. Non-compartmental analysis (NCA) was performed for calculation of PK parameters (WinNonlin version 5.2, Pharsight, United States).

## 3 Results

### 3.1 Patients

Among the 11 enrolled patients, 10 completed the study and 1 withdrew informed consent 3 days after the first dose was given (Supplementary Figure S1). All 11 patients were included in the analysis of PK, PD and safety parameters. Descriptive statistics of demographics and baseline clinical characteristics are listed in Table 1. The median (range) age of all 11 patients was 57 (46, 68) years, with the majority being male. All patients were categorized as Child-Pugh B, with 8 (72.7%) having ascites.

**TABLE 1:** Summary of demographic parameters and baseline clinical characteristics of patients with liver cirrhosis.

Characteristics	Tolvaptan (n = 11)
Age (years)	
Mean ± SD	55.4 ± 6.6
Median (Min, Max)	57 (46, 68)
**Gender, n (%)**	
Male	9 (81.8)
Female	2 (18.2)
**Height (cm)**	
Mean ± SD	168.3 ± 6.1
Median (Min, Max)	170 (158, 178)
**Weight (kg)**	
Mean ± SD	62.7 ± 10.7
Median (Min, Max)	61 (48, 77)
**Child–Pugh B cirrhosis, n (%)**	11 (100)
Etiology of liver cirrhosis, n (%)	
Hepatitis B	6 (54.6)
Alcoholic hepatitis	3 (27.3)
Others	2 (18.2)
**Accompanied with ascites, n (%)**	8 (72.7)
Child-Pugh ascites score, n (%)	
1	3 (27.3)
2	7 (63.6)
3	1 (9.1)
**Serum sodium (mmol/L)**	
Mean ± SD	139.1 ± 2.5
Median (Min, Max)	140.0 (134.0, 142.0)
**Serum potassium (mmol/L)**	
Mean ± SD	4.0 ± 0.3
Median (Min, Max)	4.0 (3.5, 4.5)
**Serum albumin (g/L)**	
Mean ± SD	29.3 ± 3.8
Median (Min, Max)	28.4 (22.9, 36.1)
**Serum bilirubin (µmol/L)**	
Mean ± SD	81.6 ± 27.0
Median (Min, Max)	84.0 (31.6, 135.2)
**Total bilirubin (µmol/L)**	
Mean ± SD	37.9 ± 20.9
Median (Min, Max)	29.5 (15.4, 73.3)
**ALT (U/L)**	
Mean ± SD	43.0 ± 21.0
Median (Min, Max)	39.0 (26.0, 98.0)
**AST (U/L)**	
Mean ± SD	54.6 ± 22.8
Median (Min, Max)	48.0 (33.0, 115.0)
**Prothrombin time (s)**	
Mean ± SD	16.7 ± 1.5
Median (Min, Max)	17.0 (14.3, 19.1)

Note. All data are presented as the mean ± SD, median with range (minimum, maximum) or as numbers with percentages.

Abbreviations: ALT, alanine transaminase; AST, aspartate transaminase; SD, standard deviation.

### 3.2 PK outcomes

**FIGURE 1:**
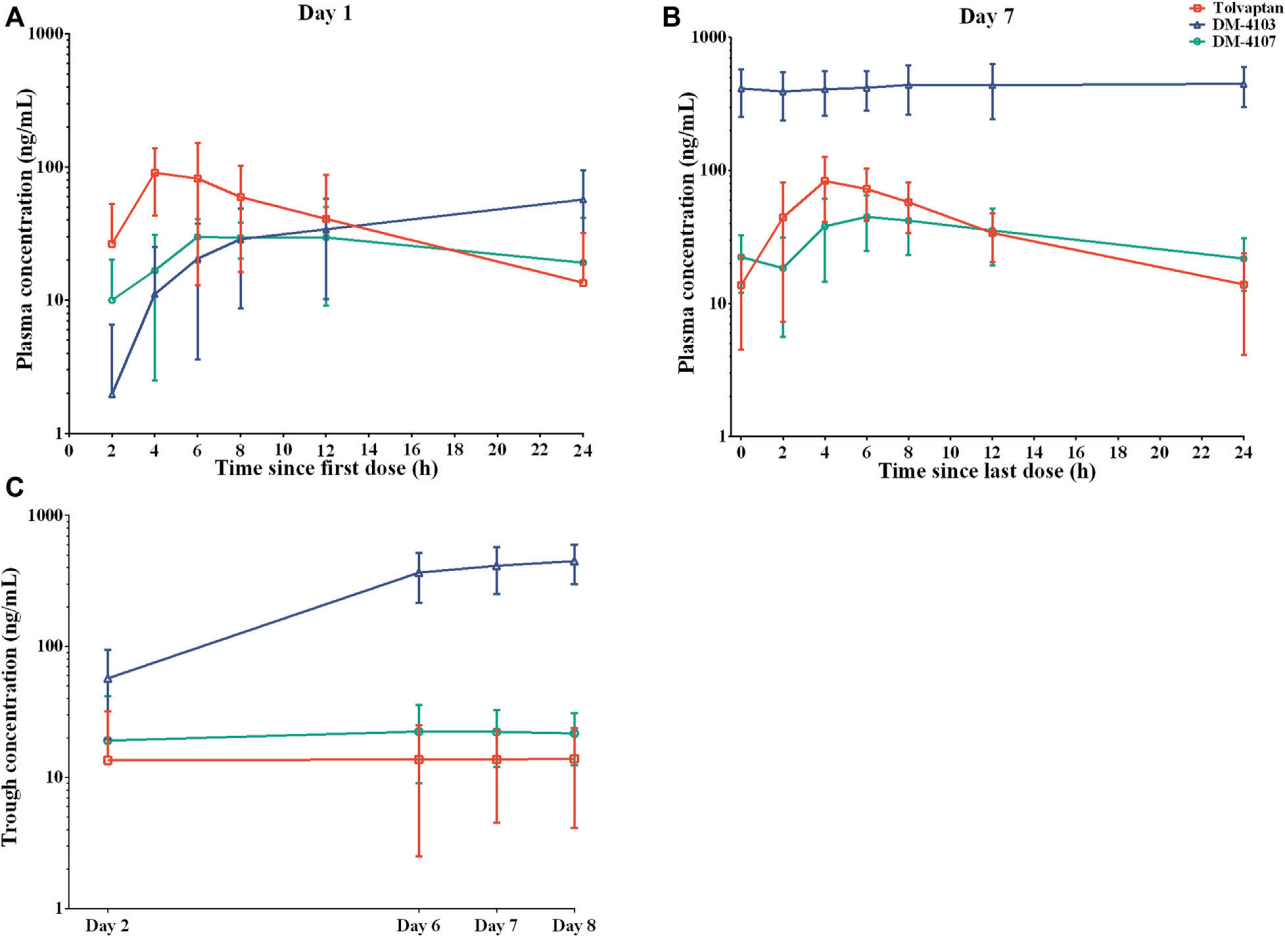
Mean plasma concentration-time profiles of tolvaptan, DM-4103 and DM-4107 after oral dosing of 15-mg tolvaptan on a semi-log scale. **(A)** Day 1 and **(B)** Day 7. **(C)** The mean trough concentration of tolvaptan, DM-4103 and DM-4107 at 24 h after 1-day, 5-day, 6-day and 7-day oral administration.

The mean plasma concentration-time curves of tolvaptan, DM-4103 and DM-4107 after 1-day and 7-day oral administration are presented in Figure 1, with PK parameters given in Table 2. After 4 h of single-dose and multiple-doses of tolvaptan, the mean plasma concentration of tolvaptan reached a maximum value of 106.0 ng/mL and 96.2 ng/mL on Day 1 and Day 7, with a t_1/2_ of 5.5 h and 6.2 h, respectively. The mean values of trough concentrations for tolvaptan and DM-4107 did not change much after 24 h dosing on Day 1, Day 5, Day 6 and Day7, ranging from 13.5 to 13.9 ng/mL and 19.1–22.4 ng/mL, respectively, while the trough concentration of DM-4103 varied greatly, ranging from 57.0 to 448.0 ng/mL. After multiple-doses of 15 mg tolvaptan, the mean R_AC_ of AUC for tolvaptan and DM-4107 were 1.3 and 1.8, respectively, while the other metabolite DM-4103 accumulated to 18.2-fold on Day 7. These results suggested that the plasma concentrations of tolvaptan and DM-4107 reached steady-state after 7 consecutive days of oral administration. DM-4103 exhibited obvious accumulation, but tolvaptan and the metabolite DM-4107 did not.

**TABLE 2:** Pharmacokinetic parameters in patients with hepatocirrhosis after a single dose and multiple doses of 15-mg tolvaptan.

PK parameters	Day 1 (n = 11)	Day 7 (n = 10)
**Tolvaptan**		
C_max_ (ng/mL)	106.0 ± 61.3	96.2 ± 32.8
AUC_0–24h_ (h·ng/mL)	939.0 ± 776.1	907.0 ± 359.5
AUC_0-t_ (h·ng/mL)	939.0 ± 776.1	907.0 ± 359.5
AUC_0-∞_ (h·ng/mL)	818.0 ± 455.7	1030.0 ± 511.1
t_1/2_ (h)	5.5 ± 1.5	6.2 ± 1.5
t_max_ (h)	4 (2, 7.98)	4 (2, 8)
CL/F (L/h)	19.4 ± 9.2	18.2 ± 5.3
V_z_/F (L)	193.0 ± 135.6	234.0 ± 190.9
R_AC_ (AUC_24h_)	-	1.3 ± 0.5
R_AC_ (C_max_)	-	1.2 ± 0.5
R_AC_ (C_24h_)	-	1.9 ± 0.9
**DM-4103**		
C_max_ (ng/mL)	58.0 ± 38.1	473.0 ± 192.9
AUC_0–24h_ (h·ng/mL)	765.0 ± 427.3	10300.0 ± 3958.9
AUC_0-t_ (h·ng/mL)	765.0 ± 427.3	10300.0 ± 3958.9
t_max_ (h)	24 (12, 24)	17.5 (0, 24)
R_AC_ (AUC_24h_)	-	18.2 ± 9.9
R_AC_ (C_max_)	-	10.9 ± 4.3
R_AC_ (C_24h_)	-	10.6 ± 4.1
**DM-4107**		
C_max_ (ng/mL)	37.3 ± 18.5	47.4 ± 20.9
AUC_0–24h_ (h·ng/mL)	528.0 ± 330.3	746.0 ± 321.9
AUC_0-t_ (h·ng/mL)	528.0 ± 330.3	746.0 ± 321.9
t_max_ (h)	6 (6, 12)	6 (3.97, 8)
R_AC_ (AUC_24h_)	-	1.8 ± 0.8
R_AC_ (C_max_)	-	1.5 ± 0.6
R_AC_ (C_24h_)	-	1.9 ± 0.8

^a^ Note. All data are presented as the mean ± SD, or median with range.

Abbreviations: AUC, area under the plasma concentration-time curve; CL/F, apparent clearance; R_AC_, accumulation ratio; t_1/2_, terminal half-life; t_max_, time to reach C_max_; V_z_/F, apparent volume of distribution.

### 3.3 PD outcomes

**FIGURE 2:**
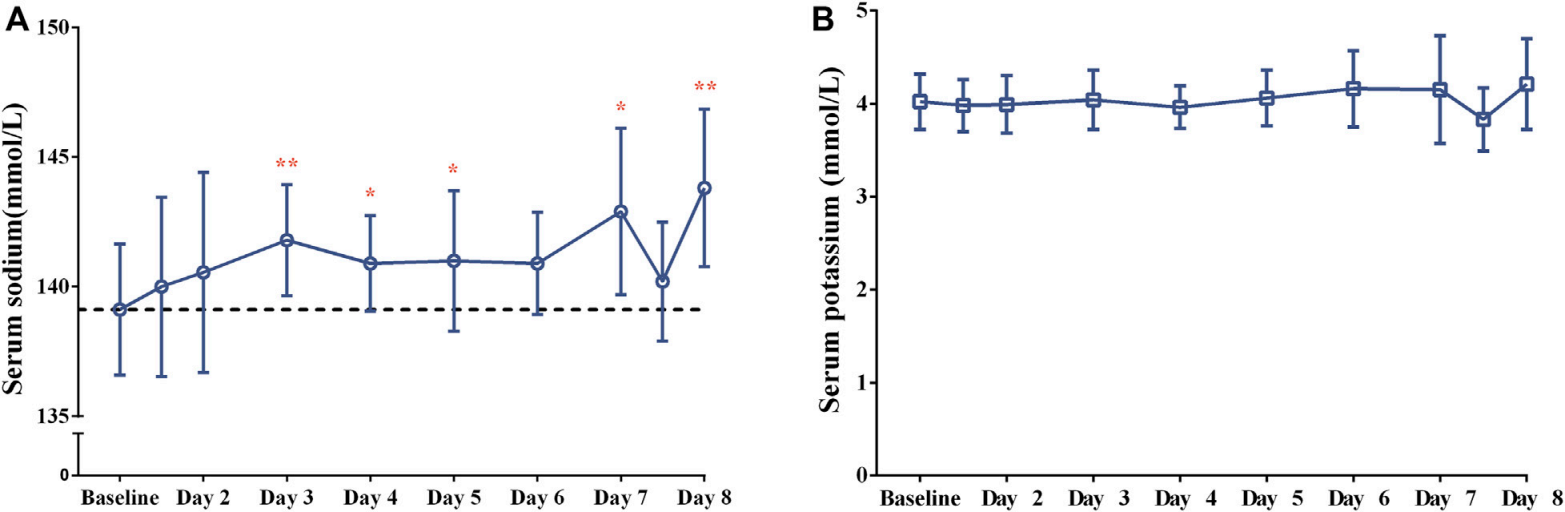
The changes of **(A)** serum sodium and **(B)** serum potassium concentrations after 7-day consecutive oral administration of 15-mg tolvaptan.

**FIGURE 3:**
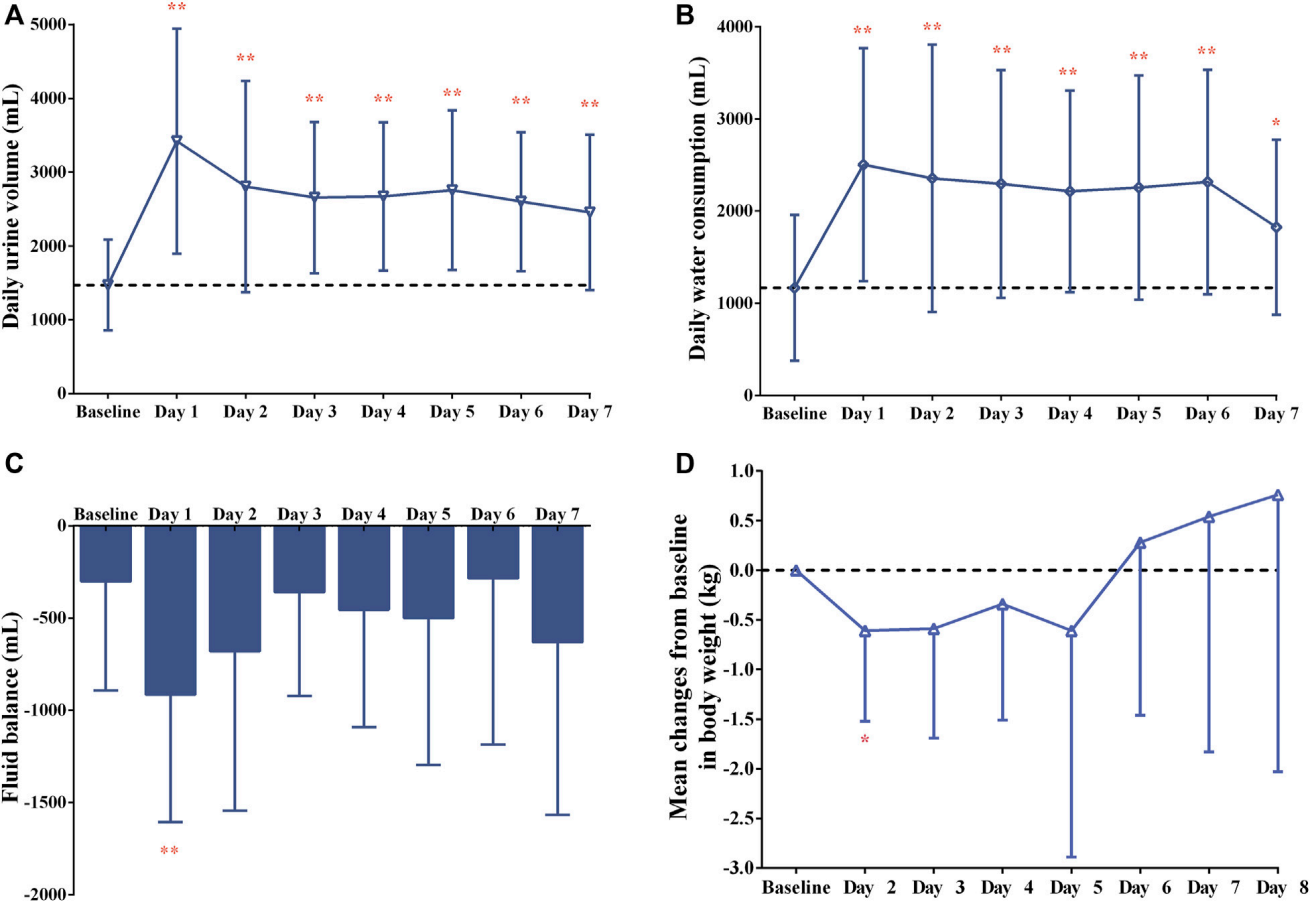
Changes in **(A)** daily urine volume, **(B)** daily water consumption, **(C)** fluid balance over time and **(D)** mean changes from baseline in body weight after 7-day oral administration of 15-mg tolvaptan.

After a single dose of tolvaptan, there was an increased trend of the serum sodium concentration, and a significant change from baseline was measured on Day 3, Day 4, Day 5, Day 7 and Day 8 (all *p* < 0.05), with the largest mean change being 4.8 ± 4.2 mmol/L on Day 8, from 139.1 ± 2.5 to 143.8 ± 3.0 mmol/L (Figure 2). However, the potassium concentration did not significantly change from baseline (all *p* > 0.05). The daily urine volume and daily water consumption were statistically significantly increased after a single-dose of tolvaptan from Day 1 to Day 7 (all *p* < 0.05), with maximum changes of 1,949.1 ± 1,251.3 mL and 1,336.4 ± 896.7 mL, respectively, both on Day 1 (Figure 3). Furthermore, patients developed negative fluid balance after 7-day oral administration of tolvaptan, with the greatest value being −612.7 ± 937.0 mL on Day 1 (*p* = 0.03, vs. baseline). Except for a pronounced decrease of body weight observed after 1-day oral administration of tolvaptan (Δ = 0.61 kg, *p* = 0.05), the trend of weight loss was still unapparent during 7 consecutive days of oral administration (all *p* > 0.05 vs. baseline).

### 3.4 Safety

Of 11 patients, 9 (81.8%) experienced 20 TEAEs, and 7 (63.6%) experienced 12 drug-related TEAEs. The most commonly occurring TEAEs were thirst (5, 45.5%), pollakiuria (4, 36.4%) and dry mouth (3, 27.3%) (Table 3), which were both associated with tolvaptan administration. The majority of TEAEs were mild to moderate in severity, with only 1 case of severe dyspnea, which was not associated with tolvaptan administration. No SAEs occurred, and no patients withdrew from the study due to TEAEs. During the whole treatment period, there were no obvious changes in the indicators from laboratory tests, vital signs and 12-ECG, and no associated TEAEs occurred.

**TABLE 3:** Summary of treatment-emergent adverse events.

	Tolvaptan (n = 11)	
	Number of events	n (%)
**Any TEAEs**	20	9 (81.8)
Mild	15	7 (63.6)
Moderate	4	3 (27.3)
Severe	1	1 (9.1)
**Drug-related TEAEs**	12	7 (63.6)
Mild	9	5 (45.5)
Moderate	3	3 (27.3)
Severe	0	0
**Any SAEs**	0	0
**Any TEAEs reported in ≥ 2 patients, termed by PT**		
Thirst	5	5 (45.5)
Pollakiuria	4	4 (36.4)
Dry mouth	3	3 (27.3)
Dyspnea	2	2 (18.2)

Abbreviations: PT, preferred term; SAE, serious adverse event; TEAE, treatment emergent adverse event.

## 4 Discussion

This study evaluated the PK, PD and safety profiles of tolvaptan after a single- and multiple-dose 15-mg oral dose in Chinese patients with Child-Pugh B cirrhosis. Regarding the design of 7-day tolvaptan medication duration in treatment of ascites previously published Chinese and Japanese studies (Sakaida et al., 2012; Wang et al., 2018; Tang et al., 2020), the medication duration of tolvaptan in the present pilot study was also designed to be continuous for 7 days, which also adhering to a continuous medication duration of no more than 30 days as specified in Chinese guidelines (Chinese Society of Hepatology and Chinese Medical Association, 2023). The plasma concentration of tolvaptan reached a maximum value 4 h after a single-dose, with a t_1/2_ of 5.5 h. The plasma concentration of tolvaptan and DM-4107 reached steady-state concentrations after 7 consecutive days of oral administration. No accumulation of tolvaptan and DM-4107 were detected, but DM-4103 accumulated to 18.2-fold on Day 7, as previously reported (Kim et al., 2011; Sakaida et al., 2012). DM-4103 is an inhibitor of organic anion transport polypeptide (OATP)1B1 and organic anion transporter (OAT)3, with a plasma half-life of 180 h (Otsuka, 2022). A previous study has shown that DM-4103 produced no clinically meaningful changes in concentrations of rosuvastatin (OATP1B1 substrate) or furosemide (OAT3 substrate) (Shoaf et al., 2021).

The t_1/2_ exhibited a dose-dependent trend in previous studies in healthy subjects and cirrhosis patients (Kim et al., 2011; Yi et al., 2012), but a dose-dependent trend was not observed in cirrhosis patients (Sakaida et al., 2012). A population PK study showed that cirrhosis also significantly affected tolvaptan PK parameters, with cirrhotic patients (Child-Pugh score ≥ 6) expected to have a 58.0% reduced CL/F and a 64.8% increased V_z_/F relative to healthy subjects (Van Wart et al., 2013). However, the increase in t_1/2_ and plasma tolvaptan concentration in cirrhotic patients was more likely to be attributed to reduced liver perfusion or metabolic capacity (lower CL/F) rather than the increases of V_z_/F. Patients with Child-Pugh B and C cirrhosis were both included in a Japanese clinical trial, while the Chinese patients in the present study only included Child-Pugh B cirrhosis; these differences may have resulted in the t_1/2_ in Japanese patients after 7.5 mg tolvaptan being about 3 h longer than in Chinese patients given 15 mg tolvaptan. In addition, a former study demonstrated that the PK characteristics in Japanese subjects were not markedly different from Caucasian individuals (Kim et al., 2011). Therefore, the differences in AUC and C_max_ might be a result of a reduced hepatic function in Japanese patients rather than due to racial and ethnic variations, an issue that warrants further studies.

After 7-day multiple-dose administration of tolvaptan, a significant change of the serum sodium concentration from baseline was found on Day 3, Day 4, Day 5, Day 7 and Day 8 (all *p* < 0.05), with the largest mean change being 4.8 mmol/L on Day 8 from baseline, as previously mentioned (Okita et al., 2010). Similar to findings of previous studies (Sakaida et al., 2012; Wang et al., 2018; Tang et al., 2020), there was a statistically significant increase in daily urine volume and water consumption from Day 1 to Day 7 after tolvaptan administration, showing a trend of negative fluid balance in the present study. Although weight losses from baseline ranged from −0.61 to −0.34 kg after 1–4 days administration of tolvaptan, a pronounced weight loss from baseline was only measured after 1-day administration (*p* = 0.05). Given that the previous two studies conducted in Chinese patients reported a significant weight loss after 7-day administration of 15 mg-tolvaptan (Wang et al., 2018; Tang et al., 2020), the lack of a statistically significant difference in weight loss may be attributed to the small sample size and individual variations of patients in the present study. Overall, tolvaptan exerted a promising diuretic effect in Chinese patients with Child-Pugh B cirrhosis after daily oral doses of 15 mg tolvaptan, which warrants further investigation with a large-scale sample size study.

Consistent with previous Chinese studies (Wang et al., 2018; Tang et al., 2020), the most common TEAEs were pollakiuria, thirst and dry mouth after consecutive administration of tolvaptan 15 mg daily for 7 days. The majority of TEAEs were mild to moderate in severity, and only 1 case of severe dyspnea occurred, which was not associated with tolvaptan treatment. There was no obvious change in liver injury-related indicators and no related TEAEs, results similar to those found in the study by Watkins et al. (Watkins et al., 2015). All in all, a lower-dose of tolvaptan (15 mg/d) for a short period (7 days) administration was well tolerated in patients with Child-Pugh B cirrhosis. The limitations of the present pilot study were the absence of a control group, a small sample size and a short treatment duration (7 days); a well-calculated and statistically well powered clinical trial will be conducted in the near future.

In conclusion, the plasma concentration of tolvaptan reached steady-state after 7 consecutive days of oral administration and no accumulation was found. Tolvaptan at 15 mg significantly increased free water excretion without significantly affecting electrolyte loss. It was well tolerated by Chinese patients with Child-Pugh B cirrhosis (accompanied by ascites), providing a promising short-term treatment regimen for ascites in these patients.

## Data Availability

The raw data supporting the conclusion of this article will be made available by the authors, without undue reservation.
