# Supplementary material for: Pharmacokinetics, pharmacodynamics and safety of 15 mg-tolvaptan administered orally for 7 consecutive days to Chinese patients with child-Pugh B cirrhosis
Source: Front Pharmacol. 2024 Jan 26;15:1324299. doi: 10.3389/fphar.2024.1324299 (PMC10853469; doi:10.3389/fphar.2024.1324299)
Supplement: Supplementary file 1 [file Image1.pdf]

Supplementary Material

1 Supplementary Figures and Tables

1.1 Supplementary Figures

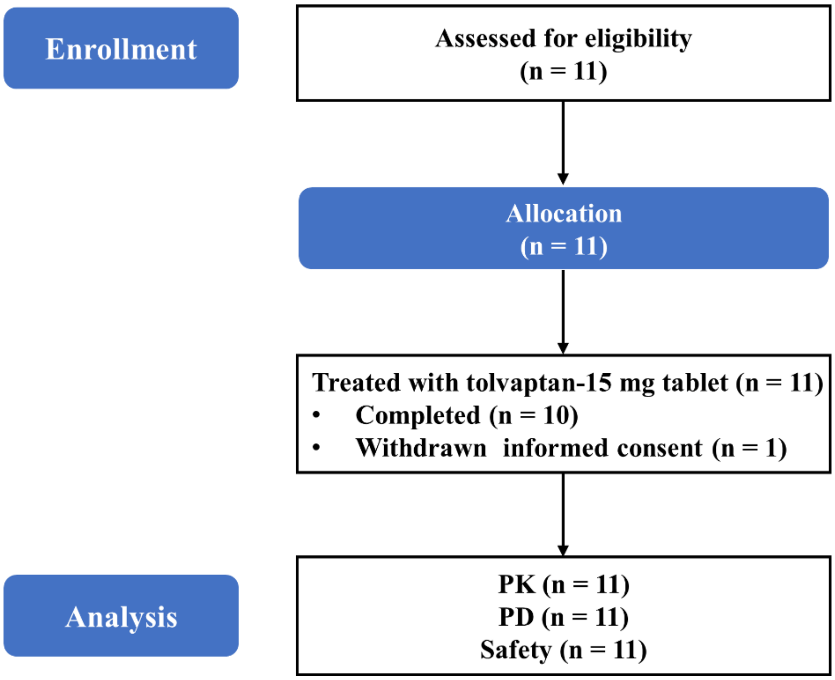

Supplementary Figure S1. Flow chart of patient disposition.
